# Controlling the Wetting Properties of Superhydrophobic Titanium Surface Fabricated by UV Nanosecond-Pulsed Laser and Heat Treatment

**DOI:** 10.3390/nano8100766

**Published:** 2018-09-27

**Authors:** The-Hung Dinh, Chi-Vinh Ngo, Doo-Man Chun

**Affiliations:** 1 School of Mechanical Engineering, University of Ulsan, Ulsan 44610, Korea; dinhthehung@mail.ulsan.ac.kr (T.-H.D.); ngochivinh@gmail.com (C.-V.N.); 2 Changchun Institute of Optics, Fine Mechanics and Physics, Chinese Academy of Sciences, Changchun 130033, China; 3 The Institute of Optics, University of Rochester, Rochester, NY 14627, USA

**Keywords:** superhydrophobic, nanosecond pulsed laser, laser power, isotropicity and anisotropicity, pattern design

## Abstract

In this study, the effects of nanosecond-pulsed laser and pattern design were researched on the wettability of titanium material. Nanosecond-pulsed laser and heat treatment are used to fabricate superhydrophobic titanium surfaces. The effects of laser power (1–3 W) and step size (50–300 µm) on a microscale patterned titanium surface (line pattern and grid pattern) were investigated to explain the relation between microstructure and superhydrophobicity. The surface morphologies and wettability of the surfaces were analyzed by three-dimensional confocal microscopy and a contact angle meter. The results show that the laser power and pattern design affected the apparent contact angle (*CA*) and sliding angle (*SA*). The maximum step size, which could show superhydrophobicity with apparent *CA* > 150° and *SA* < 10°, was increased when the laser power increased from 1 to 3 W. Grid pattern showed isotropic wetting behavior, but line pattern showed both isotropic and anisotropic wetting behavior according to step size and laser power. Furthermore, when choosing the proper laser power and step size, the wetting properties of superhydrophobic surface such as lotus effect (apparent *CA* > 150° and *SA* < 10°) and petal effect (apparent *CA* > 150° and no *SA*) and isotropic/anisotropic behavior can be controlled for applications of water droplet control.

## 1. Introduction

The superhydrophobicity of a solid surface (an apparent contact angle (*CA*) bigger than 150° and a sliding angle (*SA*) smaller than 10°) is very important for functional surfaces, and it has attracted the attention of many researchers for applications such as water collection [1], self-cleaning [2], water repellence [3], antifouling [4], antibacterial surfaces [5], anticorrosion [6], anti-icing [7], and so on. Lotus leaves and butterfly wings are popular superhydrophobic surfaces in nature. Numerous studies on superhydrophobic surfaces have been reported. A superhydrophobic surface has been achieved by the fabrication of micro/nanometer-scale rough structures [8] through different methods, such as coating [9], laser texturing [10], UV irradiation [11], and so on. These techniques all require either special equipment or complex process control. Some researchers have tried only laser beam machining without any chemicals for the easy fabrication and removal of unwanted properties of chemicals on the surface. However, immediately after laser surface texturing, the surface was hydrophilic, and the surface became a superhydrophobic surface after a long time (several days or months) under ambient conditions. On the other hand, laser surface texturing for wetting modification has been extensively studied in different materials as metal [12], polymers [13] or ceramics [14]. Some researchers used laser beam machining on a titanium surface, such as a ultrashort picosecond laser [15] or a laser micromachining, to create microstructures, and then they applied a toxic chemisorption post process on these microstructures [16] or placed the microstructures in ambient air for 30 days to make the surfaces hydrophobic [10]. Previous studies have mainly focused on how to produce superhydrophobic metallic surfaces or changing the wetting behavior from hydrophilicity to superhydrophobicity on metals when using laser beam machining.

Recently, a solution combining nanosecond pulsed laser and heat treatment to prevent the usage of toxic chemicals and long fabrication time has been reported to form superhydrophobic copper [17], titanium [18], and aluminum grid-patterned surfaces [19]. However, research has focused mainly on the change in wetting behavior on only grid-patterned surfaces. The effects of pattern design and laser power on the superhydrophobicity of metal surfaces, which also plays an important role in optimization of fabrication time as well as performance of superhydrophobic surfaces in industry and manufacturing, have not been studied yet. In this research, the effect of the microstructure based on pattern design, laser power, and step size on superhydrophobicity was studied. The obtained results could provide a useful guide to select the proper laser power, step size, and pattern design for various purposes in the efficiency of process, fabrication time, and specified applications such as control of the moving direction of a water droplet with a lined pattern design.

## 2. Materials and Methods

**Figure 1:**
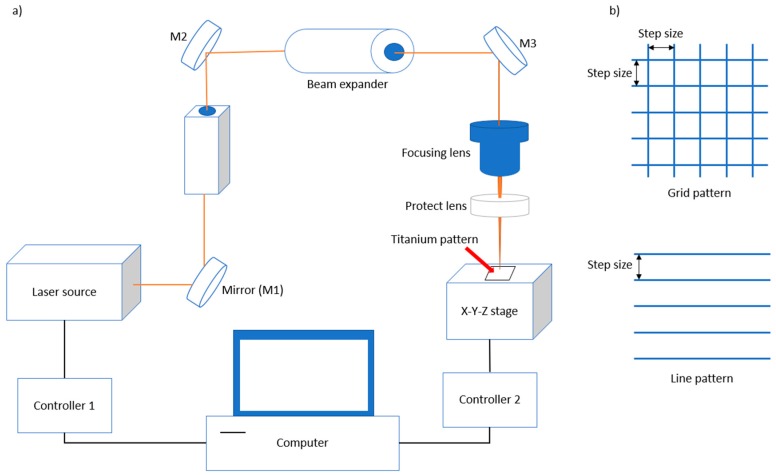
Schematic images of (**a**) the laser beam machining system; and (**b**) pattern design.

Titanium sheets (99.5% purity, Nilaco Corporation, Tokyo, Japan) with a 0.5 mm thickness were used in the experiments. A Q-switched Nd:YAG 355-nm UV nanosecond pulsed laser (Awave355-3W20K, Advanced Optowave, Ronkonkoma, NY, USA) and a focusing lens with 5 µm beam spot size were used. Figure 1a shows a schematic image of the nanosecond pulsed laser system. Laser beam machining was performed with grid and line patterns (Figure 1b), and the process parameters are summarized in Table 1. The laser power was studied from 1 to 3 W, and the step size was studied from 50 to 300 µm. Three samples for each condition were produced for reproducibility.

**Figure 2:**
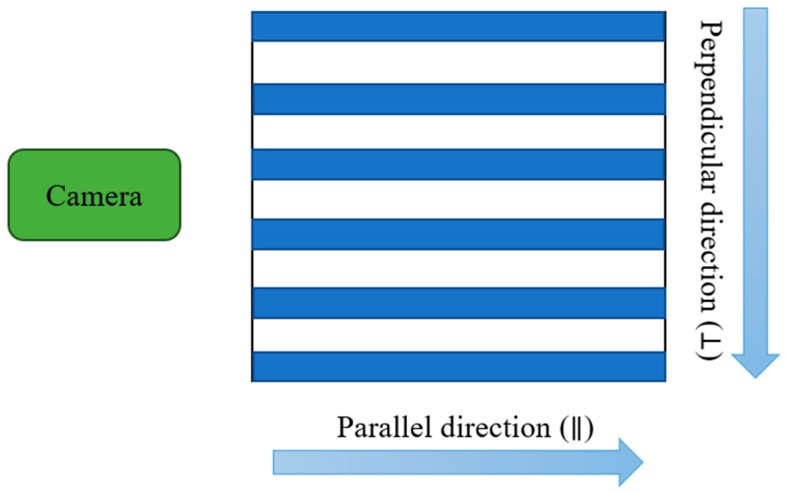
Definition of the parallel and perpendicular directions for the line pattern.

After laser beam machining, the samples were put in an oven at 200 °C for a 6 h heat treatment. The samples were then cooled naturally in ambient air for 2 h, and the apparent contact angles on samples were measured by a contact angle meter (SmartDrop SDLab-200TEZD, Femto Fab, Seongnam, Korea) to evaluate the wettability of the samples. The apparent *CA* of each sample was measured one time with an 11 µL volume of water because the water droplet could be easily placed on a titanium surface. As shown in Video S1, the sample fabricated with 1 W laser power and 50-µm step size showed low adhesion of droplet water with 10 µL volume and the water droplet could not be transferred from needle to the surface. A three-dimensional (3D) laser scanning confocal microscope (VK-X200 series, Keyence, Osaka, Japan), a field emission scanning electron microscopy (FESEM, JSM-6500F, Jeol Co., Tokyo, Japan), and energy-dispersive X-ray spectroscopy (EDS, JSM-6500F, Jeol Co., Tokyo, Japan) were used to analyze the surface structure. Commonly, a water droplet placed on the grid pattern exhibits isotropic wetting behavior, while one placed on the line pattern shows anisotropic, parallel and perpendicular directional wetting, as shown in Figure 2.

## 3. Results

### 3.1. Surface Morphology

**Figure 3:**
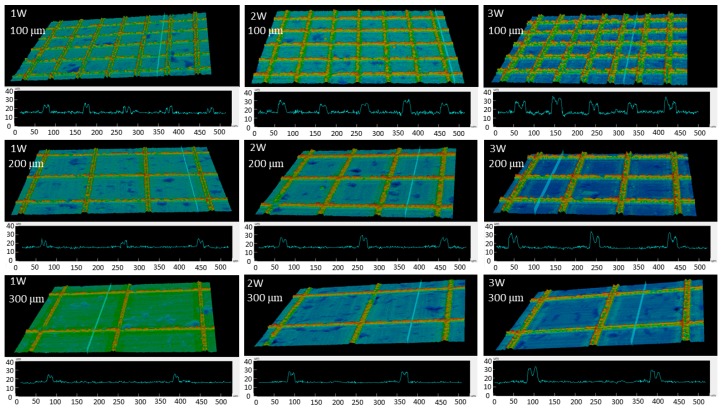
Confocal microscopy images of the grid-patterned samples with a step size of 100, 200, and 300 μm at a laser power of 1, 2, and 3 W.

**Figure 4:**
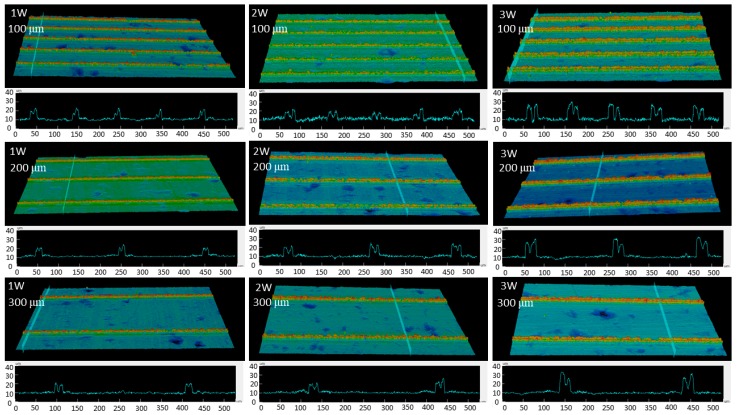
Confocal microscopy images of the line-patterned samples with a step size of 100, 200, and 300 μm at a laser power of 1, 2, and 3 W.

The two-dimensional (2D) and 3D images of the laser-machined surfaces with different laser powers, step sizes and pattern designs were observed by 3D confocal microscopy as shown in Figure 3 and Figure 4. The grid and line patterns were clearly fabricated by nanosecond pulsed laser. Nonfabricated flat areas between the grid and line patterns were also observed.

Figure 3 and Figure 4 show the typical grid and line pattern structures measured by 3D confocal microscopy. Burrs around laser machined areas were clearly observed. The height and width of the burr increased as the laser power increased. The average heights of the burrs in grid patterns were approximately 9.94 ± 1.7 µm at 1 W, 10.95 ± 0.8 µm at 2 W, and 15.81 ± 0.5 µm at 3 W, while the line patterns were approximately 9.83 ± 2.7 µm at 1 W, 12.97 ± 3.25 µm at 2 W, and 18.09 ± 3.2 µm at 3 W. The average widths were approximately 22.27 ± 4.5 µm at 1 W, 24.38 ± 0.85 µm at 2 W, and 29.22 ± 0.95 µm at 3 W for grid pattern, and those for the line patterns were approximately 18.73 ± 2.5 µm at 1 W, 25.56 ± 2.5 µm at 2 W, and 29.54 ± 3.5 µm at 3 W.

### 3.2. Wettability

**Figure 5:**
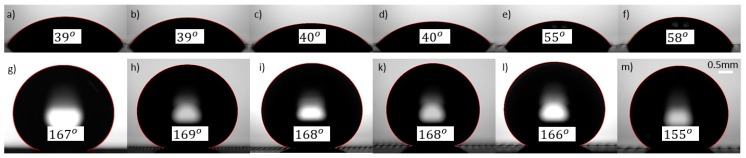
Images of water droplet apparent contact angle: (**a**–**f**) initial stages, (**g**–**m**) after heat treatment for line-patterned samples with a step size of 50 μm (**a**,**g**), 100 μm (**b**,**h**), 150 μm (**c**,**i**), 200 μm (**d**,**k**), 250 μm (**e**,**l**), and 300 μm (**f**,**m**) at 3 W with 0.5 mm scale bar for all images.

The typical images of apparent *CA* before and after heat treatment are shown in Figure 5 for line-patterned samples at 3 W laser power. Before heat treatment, all samples showed the apparent *CA*s less than 90° (hydrophilic), as shown in Figure 5a–f, but the samples became superhydrophobic surfaces after heat treatment, as shown in Figure 5g–m.

**Figure 6:**
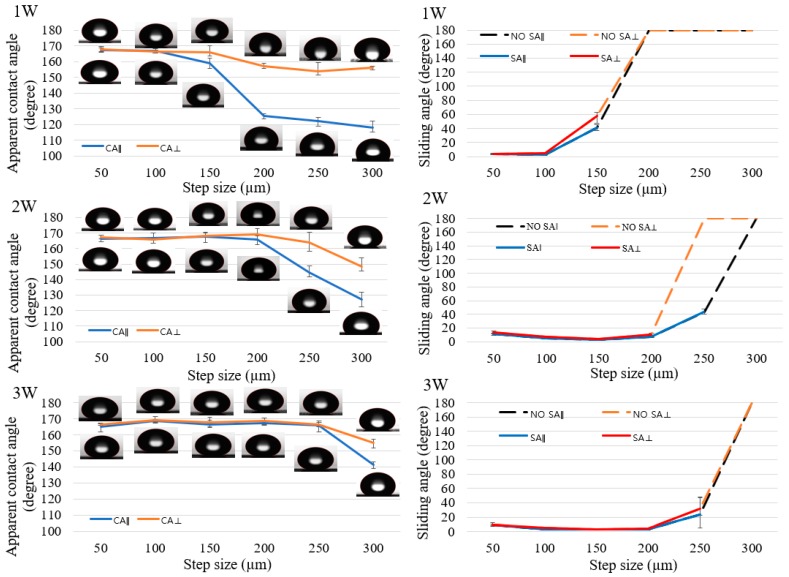
Measurement of the water droplet contact and sliding angle of line-patterned samples with two directions: parallel direction (blue color) and perpendicular direction (orange color) at laser power of 1, 2, and 3 W.

Figure 6 showed the change in wetting state on line-patterned surfaces with laser power and step size. When the laser power decreased, the critical step size also changed. The critical step size was the point where the wetting state of the surface tended to change from isotropic to anisotropic behavior. From the difference of apparent contact angles (∆*CA*) between two directions (parallel and perpendicular to the line patterns), isotropicity and anisotropicity were defined [20]. The calculation of ∆*CA* was performed using the following equation:

(1) $$\Delta CA=\left|CA_{⊥}-CA_{∥}\right|$$

If ∆*CA* < 10°, then the material is called isotropic; if ∆*CA* > 10°, then it is called anisotropic. When the laser power increased from 1 W to 2 W and then to 3 W, the critical step size changed from 150 µm to 200 µm and then to 250 µm for the apparent contact angle difference, respectively, as shown in Figure 6. The laser power at 3 W did not show any differences between the parallel direction and perpendicular direction for all step sizes smaller than 250 µm. At a 300-µm step size of line-patterned samples with 3 W, the apparent contact angles following the parallel direction and perpendicular direction showed a clear difference. At 2 W with a 250-µm step size, there was a clear difference in apparent *CA* between the two directions, as there was at 1 W with a 200-µm step size. The more the step size increased, the larger was the difference between the apparent *CA* of the two directions. Decreasing laser power did not have an effect at a small step size (especially at 50 and 100 µm); however, at a large step size (from 150 to 300 µm in this research), the difference between the parallel and perpendicular directions was large, especially at a 300-µm step size for all laser powers. Additionally, the anisotropic behavior was clear in the sliding angle results. For example, with a laser power of 2 W at 250-µm step size, the sliding angle exhibited along the parallel direction but did not show along the perpendicular direction. The apparent contact angles and sliding angles following the perpendicular direction were always greater than those following the parallel direction. Following the parallel direction, a water droplet can easily move on the surfaces because there is no barrier along the moving direction of the water droplet; along the perpendicular direction, the burr acted as a barrier, which prevented the movement of the water droplet, and the water droplet was more difficult to move than along the parallel direction.

**Figure 7:**
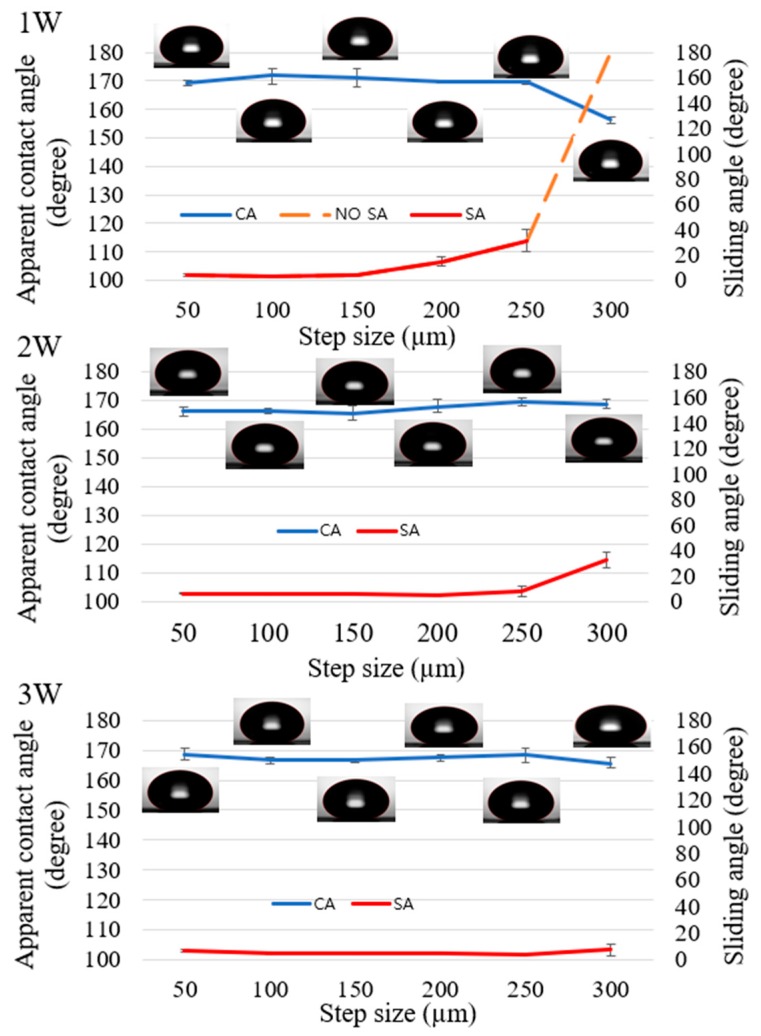
Measurement of apparent contact angle (blue color) and sliding angle (red color) of grid-patterned samples at laser power of 1 W; 2 W; and 3 W.

Figure 7 shows the apparent contact angle and sliding angle for the grid pattern. Similar to line-patterned samples, when the laser power changed, the critical step size, where the wetting state tended to change, also changed. The grid-patterned samples included two critical step sizes where the “lotus effect” wetting state, which has apparent *CA* > 150° and *SA* < 10°, changed to the wetting state which has apparent *CA* > 150° and *SA* > 10°, and the wetting state, which has apparent *CA* > 150° and *SA* > 10°, changed to the “Petal effect” wetting state which has apparent *CA* > 150° and no *SA*. From the values of sliding angle, the wetting state of the grid-patterned samples was defined. If the sliding angle was smaller than 10°, the surface showed lotus effect. If the sliding angle was greater than 10°, the surface might not show any lotus effect or petal effect. The surface showed the petal effect when there was no sliding angle. In Figure 7, when the laser power increased from 1 to 2 W, the critical step size, where the wetting state changed from the lotus effect wetting state, changed from 150 to 250 µm. At 2 and 3 W, all step sizes showed an apparent contact angle of approximately 165° and sliding angle smaller than 10°, except at the 300-µm step size at 2 W, the SA was 32°. At 1 W with a 300-µm step size, there was no SA, and apparent *CA* was 157 ± 1.3°. At 200-µm and 250-µm step size for 1 W, the SA was greater than 10°.

**Figure 8:**
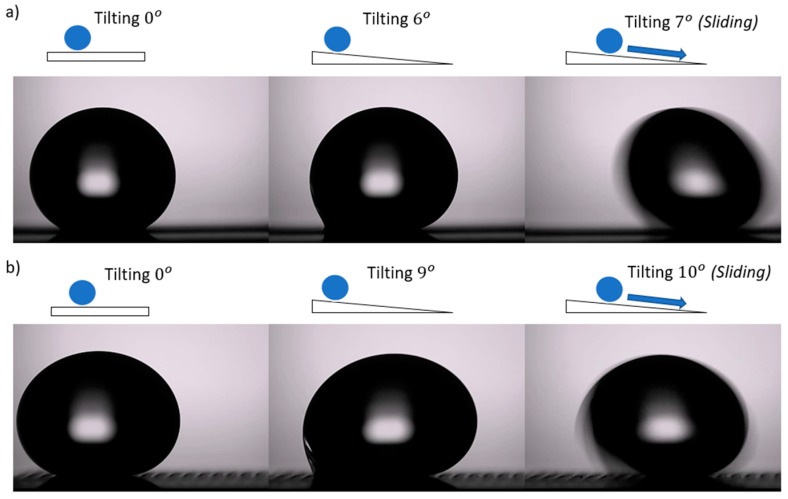
Sliding angle of the line-patterned sample at laser power 3 W with a 200-μm step size: (**a**) parallel direction, and (**b**) perpendicular direction.

The images of sliding angle on the titanium surfaces were shown in Figure 8 for line patterns of a 200-μm step size at 3 W laser power. The substrate was tilted at a speed of 1.6°/s, and the water droplet started sliding at approximately 7° for the parallel direction and 10° for the perpendicular direction. In this study, several step sizes did not have a sliding angle when tilting to 90°; even when tilting manually to 180°, the water droplet did not leave the surface. The water droplet might contact the hydrophilic surface and show the strong attraction to this surface. Therefore, the water droplet cannot move off the surface. To illustrate the case where the surfaces do not have a sliding angle, we assume that their sliding angles approach 180°.

## 4. Discussion

### 4.1. Mechanism

**Figure 9:**
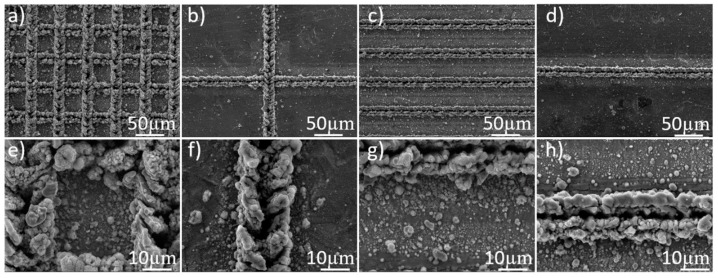
Top-view (**a**–**d**) field emission scanning electron microscopy (FESEM) images and (**e**–**h**) enlarged images of laser-machined surfaces with different step sizes 50, 300 µm for grid pattern, and 50, 300 µm for line pattern, respectively.

The phenomena of wetting transition on the titanium surfaces from hydrophilic become superhydrophobic surface after heat treatment time could be interpreted by investigation of surface chemistry as well as surface morphology. The surface structures of titanium were nano-micro hierarchical structure after laser beam machining and there was no clear change before and after heat treatment. The fabricated paths make clearly structure on titanium surface as show in Figure 3, Figure 4 and Figure 9. The superhydrophobicity on titanium surface was increased as heat treatment time was increased. The results of EDS showed that the atomic ratio of elements on the burr was changed before and after the heat treatment as shown in Table 2. After heat treatment, wettability of all samples changed from hydrophilic surface to superhydrophobic surface. From the results of EDS, the amount of carbon content on the burr was increased regardless of step size and pattern. This result is similar to other researcher’s results. The mechanism was reported as organic absorption of hydrophobic groups (–CH_3_) [10,21,22]. And this organic absorption can happen in the air, but heat treatment can accelerate the organic adsorption. Therefore, nano-micro hierarchical structures by laser beam machining and low energy surface by organic adsorption could make the surface superhydrophobic.

### 4.2. Effects of Laser Power and Step Size on Wettablity

Increasing the laser power from 1 to 3 W and changing the step size from 50 to 300 µm had an effect on the surface wettability of the grid-patterned surface and the line-patterned surface. The height and width of the burr increased when increasing the laser power from 1 to 3 W. The dimension of microburrs affected the wettability as well as the critical step size. At a small step size (examples: 50, 100 µm), microburrs still supported the water droplet, but at a big step size (200, 250, 300 µm), the water droplet might penetrate between the microburrs, and the water droplet could touch on the flat surface. Therefore, a pinning effect was observed, which resulted in isotropic to anisotropic behavior transition of the line-patterned surfaces and the SA change of the grid-patterned surfaces.

**Figure 10:**
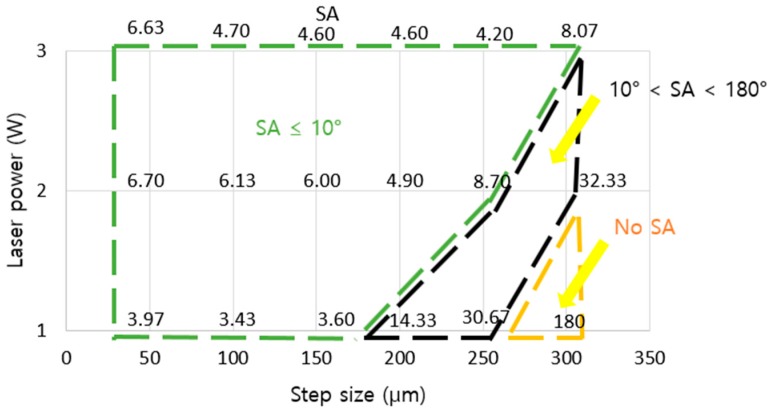
Effect of grid-patterned microstructure on the wetting transition with SA.

The apparent contact angles of all samples were greater than 160°, except samples at 1 W laser power with 300-μm step size. The burr height at 1 W laser power was the lowest and the step size of 300 µm was the biggest. Therefore, the water droplet could not be supported by burrs and it affected the apparent contact angle and sliding angle. The variation of the sliding angle of the grid-patterned samples showed a clear effect on the decrease in laser power. The values of the sliding angles can be divided into three regions (*SA* ≤ 10°, 10° < *SA* < 180°, and no *SA*) as shown in Figure 10 and Video S2 demonstrate typical *SA*s from samples with three different step sizes 50, 250 and 300 µm at 1 W laser power for three regions. This result is helpful for other researchers to choose a proper laser power and step size to fabricate the desired superhydrophobic surfaces for specific applications.

With the line pattern, the apparent contact angle decreased when the laser power changed from 3 to 1 W while the sliding angle increased and did not have a sliding angle at a large step size (200–300 µm). At a small step size (50–100 µm), when decreasing the laser power, the values of the apparent contact angle and sliding angle show small difference. When decreasing the laser power with the line samples, the apparent contact angle showed clearly anisotropic behavior at a large step size (200–300 µm at 1 W, 250–300 µm at 2 W and 300 μm at 3 W). These results could provide a useful guide to select the proper values of laser power, step size, and pattern design to produce an apparent contact angle larger than 160° and a sliding angle smaller than 10°.

**Figure 11:**
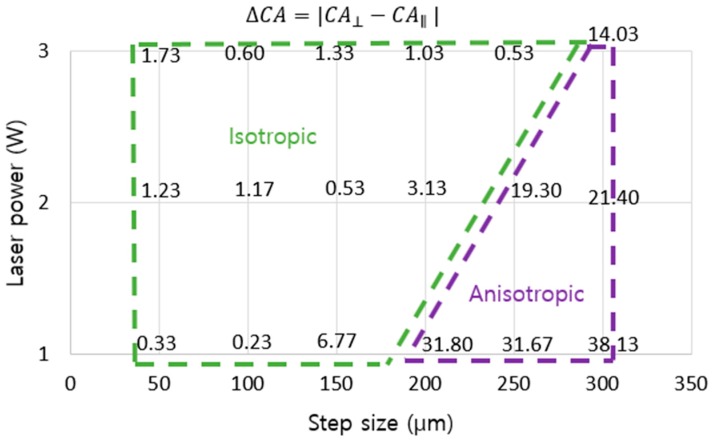
Effect of line-patterned microstructure on the wetting transition from the isotropic state to the anisotropic state.

With the same laser power, the grid-patterned samples showed apparent *CA*s and *SA*s better than the line-patterned samples. For good superhydrophobicity and isotropicity, the grid pattern was better. However, for control of water direction applications, the line pattern can be utilized more effectively. The laser power and step size show a clear change in the anisotropy in Figure 11. Figure 11 shows two regions with Δ*CA* < 10° and Δ*CA* > 10° related to the isotropic wetting state and anisotropic wetting state, respectively. It is a useful guide for selecting the isotropicity or anisotropic wetting state based on the laser power and step size values. In addition, SAs can be controlled by line patterns. Isotropic SA and anisotropic SA were shown in Videos S3 and S4 at 150 and 250-µm step sizes with a laser power of 2 W, respectively.

### 4.3. Stability

**Figure 12:**
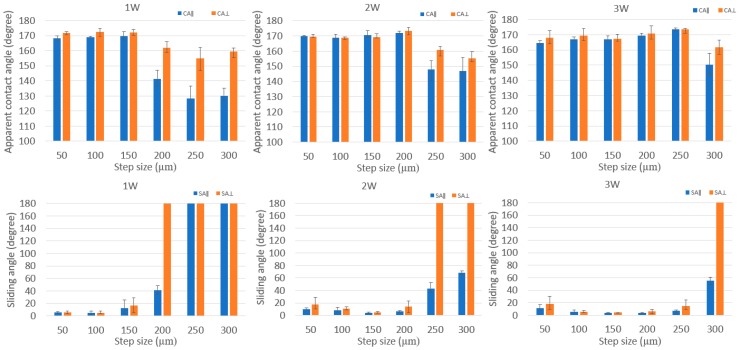
Apparent contact angle and sliding angle for the line pattern 35 days after heat treatment.

**Figure 13:**
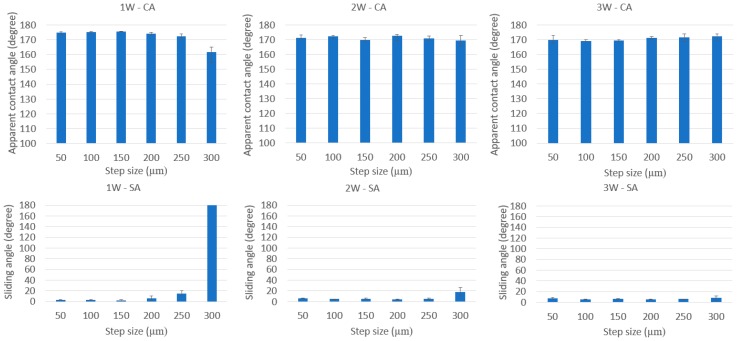
Apparent contact angle and sliding angle for the grid pattern 35 days after heat treatment.

After heat treatment, all samples were put in ambient air for 35 days. Wettability measurements were then performed again for all samples, as shown in Figure 12 and Figure 13. With the grid pattern, after 35 days, the apparent contact angle increased from 1° to 6° and sliding angle was smaller than 10°. Especially at a laser power of 1 W with a step size of 300 µm, the sliding angle was greater than 30°, which was two times larger than just after heat treatment, and the apparent contact angle increased from 156° to 162°. As shown in Figure 12, line-patterned samples showed behavior similar to the grid pattern. The apparent contact angle increased and sliding angles decreased to less than 5° or 10° at several step sizes compared to their values just after heat treatment. Of special note, several samples that had no sliding angle just after heat treatment now showed a sliding angle, indicating superhydrophobicity, with the sliding angle improving over time.

In addition, water droplets with 10 µL volume were dropped from a height of 7 cm onto these surfaces with the tilting angle of 4° and water droplet bouncing was clearly observed as shown in Video S5 and S6. This demonstrates good stability of superhydrophobic surfaces fabricated by laser beam machining and heat treatment.

## 5. Conclusions

This study developed a method to produce a superhydrophobic surface on titanium with no toxic chemicals and with a short time fabrication. The effects of microstructure and step size on superhydrophobicity were investigated. At the same laser power, the grid pattern showed better apparent *CA* and *SA* than the line pattern. When decreasing laser power in line-patterned samples, the critical step size for the isotropic to anisotropic transition region was reduced from a large to smaller step size. The anisotropic behavior was clearly observed at 1 W with a 200-µm to 300-µm step size. With decreasing laser power in the grid-patterned samples, the critical step size for the lotus effect and petal effect region was reduced. The obtained results could provide a useful guide to select proper fabrication parameters for the fabrication of desired superhydrophobic surfaces. For a high quality superhydrophobic surface and isotropicity, the grid pattern was a good candidate. To make a superhydrophobic surface with strong anisotropic behavior to control the water direction, the line pattern is preferred.

## Figures and Tables

**Table 1:** Process parameters of the laser beam machining.

Name of Parameter	Value
Power (W)	1, 2, 3
Pulse frequency (kHz)	20
Pulse duration (ns)	20
Stage speed (mm/s)	1
Step size (μm)	50, 100, 150, 200, 250, 300

**Table 2:** Energy-dispersive X-ray spectroscopy (EDS) results on burrs before and after heat treatment.

Element (Atomic %)	Line Pattern				Grid Pattern			
	Step Size 50 μm		Step Size 300 μm		Step Size 50 μm		Step Size 300 μm	
	Before	After	Before	After	Before	After	Before	After
C	6.51	7.33	6.43	9.35	5.67	7.6	7.33	8.35
O	68.26	68.51	47.95	64.52	62.96	63.62	56.95	65.94
Ti	25.23	24.16	45.62	26.12	31.37	28.79	35.72	25.71
C/Ti	0.26	0.30	0.14	0.36	0.18	0.31	0.16	0.32
O/Ti	2.71	2.84	1.05	2.47	2.01	2.63	1.25	2.52

## Supplementary Materials

The following are available online at http://www.mdpi.com/2079-4991/8/10/766/s1, Video S1: Water droplet contact behavior with volume of 10 µL on the superhydrophobic grid patterned surface fabricated with 1W laser power and 50-µm step size; Video S2: SAs on grid patterned samples fabricated with 50-, 250- and 300-µm step sizes and 1 W laser power; Video S3: SAs on line-patterned sample fabricated with 150-µm step size and 2 W laser power along two different directions; Video S4: SAs on line-patterned sample fabricated with 250-µm step size and 2 W laser power along two different directions; Video S5: Bouncing of water droplet on grid patterned sample fabricated with 50-µm step size and 1 W laser power; Video S6: Bouncing of water droplet on line-patterned sample fabricated with 100-µm step size and 1 W laser power.
